# Choice of health providers and health-seeking behaviour among forest goer population in Myanmar: findings from a cross-sectional household survey

**DOI:** 10.1186/s12936-022-04356-7

**Published:** 2022-12-14

**Authors:** May Me Thet, Myat Noe Thiri Khaing, Su Su Zin, Si Thu Thein, Kemi Tesfazghi

**Affiliations:** 1 Population Services International Myanmar, No.16, West Shwe Gone Dine 4Th Street, Bahan Township, 11201 Yangon, Myanmar; 2 Greater Mekong Subregion Elimination of Malaria Through Surveillance (GEMS+), 1120 19Th St NW #600, Washington, DC 20036 USA

**Keywords:** Forest goers, Health seeking, Febrile illness, RDT, Provider choice

## Abstract

**Background:**

In Myanmar, malaria still poses a significant burden for vulnerable populations particularly forest goers even though impressive progress has been made over the past decade. Limited evidence existed related to forest goers’ health-seeking behaviour and factors that drive decision making for providers’ choice to support national malaria programmes towards elimination. In response to that, this research is conducted to identify who they preferred and what are the factors associated with providers’ choice in malaria febrile illness and Rapid Diagnostic Testing (RDT).

**Methods:**

A cross-sectional study applying quantitative household survey was completed with 479 forest goer households in 20 malaria endemic townships across Myanmar. The household data was collected with the types of providers that they consulted for recent and previous febrile episodes. To identify the factors associated with providers’ choices, univariate and multivariate multinomial logistic regressions were done using Stata version 14.1. Statistical significance was set as p = 0.05.

**Results:**

A total of 307 individuals experienced fever within one month and 72.3% sought care from providers. Also, a total of 509 forest goers reported that they had a previous febrile episode and 62.6% received care from a provider. Furthermore, 56.2% said that they had RDT testing during these previous febrile illnesses. They consulted public facilities and public health staff, private facilities, private and semi-private providers, community health volunteers or workers in their residing village and those located outside their villages but majority preferred those within their villages. On multivariate analyses, second richest quintile (public, RRR = 12.9) (semi-private, RRR = 17.9), (outside, RRR = 8.4) and access to 4 and above nearby providers (public, RRR = 30.3) (semi-private, RRR = 1.5) (outside, RRR = 0.5) were found to be significantly associated with provider choice for recent fever episode. Similar findings were also found for previous febrile illness and RDT testing among forest goers.

**Conclusions:**

It was highlighted in this study that in forest goer households, they preferred nearby providers and the decision to choose providers seemed to be influenced by their access to number of nearby providers and socio-economic status when they sought care from a provider regardless of fever occurrence location. It was important that the national programmes considere involving these nearby providers in elimination efforts.

## Background

Although malaria cases in the Greater Mekong Sub-region (GMS) were reduced by 90% between 2000 and 2019, it remains a major disease burden in the region and is compounded by the presence of artemisinin [1]. Compared to other countries in the South-East Asia and the Greater Mekong Sub-Region (GMS), malaria incidence in Myanmar is the second highest among the GMS countries, accounting for 31% of cases in the region in 2019 [1]. Nationwide, the malaria burden reduction effort has successfully reduced malaria cases by 82% and death by 93% between 2012 and 2017. However, malaria accounted for 76,518 cases and 19 reported deaths in 2018. It was 45,756 cases and 12 deaths in 2019 and it was 58,132 cases and 10 deaths in 2020 [2, 3].

In spite of intensive malaria control activities, forest malaria remains as a major health issue in forest and forest fringe communities in Myanmar. Forests are a significant risk factor that influences disease pattern in addition to migrations and human factors [4, 5]. Thus, people living or spending time in remote forested area and working for prolonged periods in the forest are at high risk of malaria in Myanmar [6–8]. First population-based sero-prevalence study in central Myanmar identified the behavioral risk factors such as working aged men had association with endemic transmission of malaria. Hence, to facilitate malaria elimination, targeting working aged men and detecting sub-clinical infections was recommended [9]. Moreover, their habit of sleeping overnight in the forests, often without mosquito nets increased their vulnerability to be infected with malaria [10].

In Myanmar community volunteers were first used for malaria control activities in 2004, when the Myanmar Council of Churches commenced a community-based malaria control project focusing on early diagnosis and treatment in remote villages. It was recognized by the Myanmar National Malaria Control Programme and other partners working in malaria and they applied the same model throughout the country. Myanmar had successfully utilized the malaria community volunteer model, in conjunction with the government health facility-based model [11].

Although the World Health Organization (WHO) had strongly emphasized that early diagnosis and prompt treatment should occur within 24 hours of the onset of symptoms to decrease risk of severe complications and onward transmission [12], several factors influenced malaria treatment-seeking behaviour, such as socio-economic factors, client health knowledge, beliefs and access to health services [13]. In Myanmar, key challenges to effective case management were the shortage of laboratory services and proper health facilities especially in remote, isolated and inaccessible areas. In these areas, a considerably large number of populations sought treatment from private sector, without being reported to public health systems [14].Another study found that almost 50% of private providers were more likely to administer anti-malarial without any diagnostic test [15]. Moreover, it was shown in Wa Region that about 80% of patients with febrile illness sought care from the private retail sector (drug peddlers, shops and market stalls) [16]. The findings from Myanmar were consistent with studies from countries in other regions. About one third of population in the GMS Region (which amounts to approximately 7 million people) lived in remote, often hilly, and forested areas where malaria transmission was high. The majority of them were very poor, had little education, and resided in isolated villages with little or no access to basic health services [14].Studies had demonstrated that multiple treatments were common for a single episode of malaria illness in the form of self-treatment alone or either with some consultation with official health sector or village health workers at some point of their illnesses [17–19]. A systematic review of qualitative studies on behaviour and perception of forest goers from GMS countries indicated that treatment-seeking practice among these populations was highly heterogeneous and often involved multiple points of care. Moreover, malaria prevention interventions, such as health education, active case detection, bed net distributions, and delivering anti-malarial drugs to forest goers’ group were potentially complex as forest goers were often absent during these village-based interventions, having concerns about blood tests and poor adherence to treatment courses [20]. Those seeking care outside of their homes chose private health care providers for malaria treatment despite greater treatment costs because of the higher perceived quality of the services they gave [21, 22].

**Fig. 1. Study procedure:**
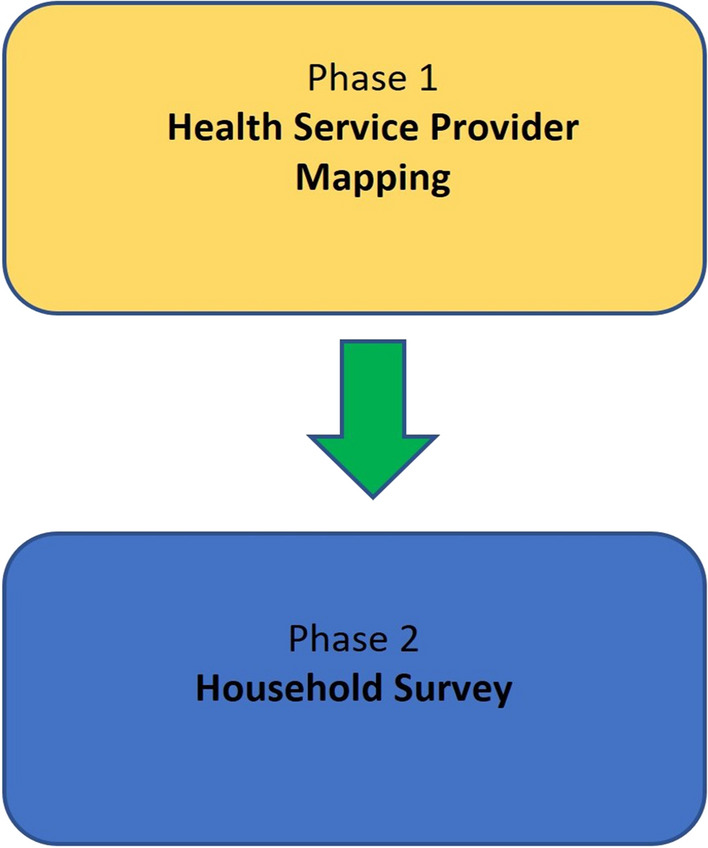
.

Nonetheless, a variety of efforts were currently undertaken in Myanmar to enhance access to malaria preventive and control measures, including ensuring equity of services accessibility irrespective of gender and race, in accordance with policies in the National Malaria Elimination Plan 2016–2030. As the policy identified the forest going population as a high-risk group for malaria, it was necessary to understand their provider preferences and influencing factors for decision in order to design appropriate and effective interventions [23]. Moreover, limited research had explored issues on forest goers in particular: how they made choices among health care providers for febrile illness, their access to health care services and the factors that influence their health-seeking decisions. The objective of this study is to explore forest goers’ health-seeking choices or febrile illnesses and malaria testing Fig. 1.

## Methods

This study was a population based cross sectional study conducted in high malaria burden areas of Myanmar in 2019.

### Study location

**Fig. 2. Map showing 20 study townships in 6 states and regions of Myanmar:**
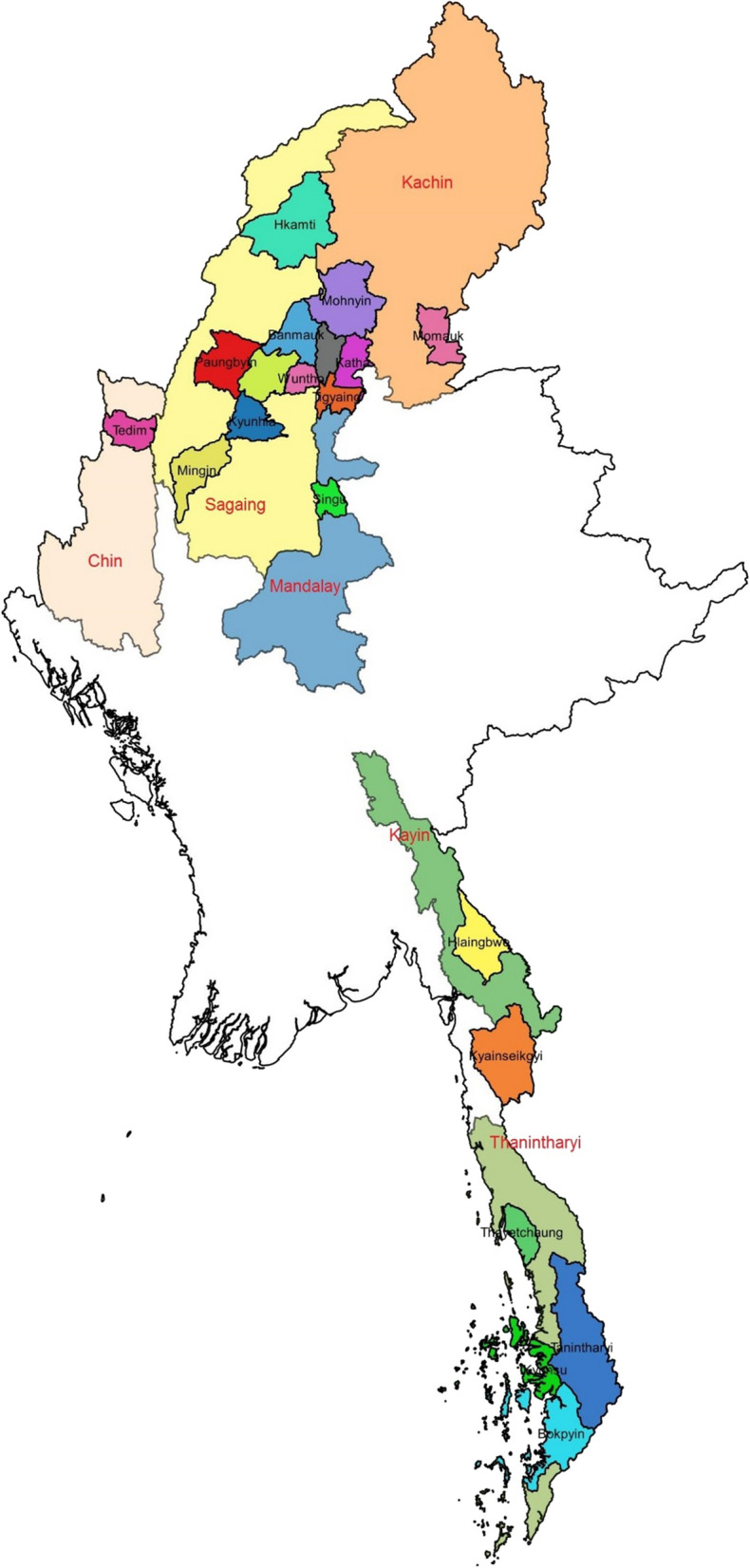
.

The study townships were 20 townships from 6 States and Regions with highest number of total malaria positive cases in 2018. They were 10 from Sagaing, 1 from Mandalay, 4 from Tanintharyi, 2 from Kayin, 1 from Chin and 2 from Kachin (Fig. 2). The study townships were the overlapping townships of the two criteria such as (1) hot spot townships, as defined by National Malaria Control Programme where the total number of positive cases was above 1000 in the year 2018, and (2) townships with at least 50 positive cases in the year 2018 as reported in PSI/Myanmar Management Information System data. Townships were the third level administrative divisions in Myanmar. A typical township consisted of urban wards and rural villages. Rural villages, primary place of residence for forest goers, were selected as primary sampling units. A total of 40 villages were selected from 20 townships where 1 village was selected from 6 townships, 2 villages were selected from 9 townships, 3 villages were selected from 4 townships and 4 villages were selected from 1 township. When selecting the villages, villages that were within 2 kms away from forest were screened first and probability-proportional-to-size (PPS) sampling was used to select the villages.

### Study procedure

The study consisted of two phases. In the first phase, a census mapping of all the health service providers (public, private, informal or traditional) in the selected villages was conducted. This was followed by a quantitative household survey in the second phase (Fig. 1).

#### Health service provider mapping

Census exercise was completed in all selected villages so as to link the available services in these villages with the health-seeking for fever episode. Upon arrival, community leaders and gatekeepers were contacted by the survey team to identify the formal and informal health services available within the selected villages. Then, a road by road census of the village was conducted to identify all the service providers and confirmed the service provider type.

#### Household survey

After mapping exercise in each selected village, random households were selected using systematic random sampling and screened for eligibility of having at least one forest goer. For the purpose of the study, forest goers were defined as: adult men or women aged 18 and above, who spent at least 1 night in the forest during the past 4 weeks. An interview was conducted in the household with at least one forest goer. Informed consent was obtained from selected participants before the interview. In the household with more than one forest goers, the one who had stayed in the forest for the longest period was selected for the interview. Among 1,680 households screened, 713 (42.4%) eligible households were visited by the study team. Total of 479, 67.2% of eligible households completed interviews where at least one household member was eligible for the interview. Eligible participants were not available at their residences for interview in 209 households, 3 households refused to participate in the study and interviews could not be completed in 22 households because they did not come back from forest for interviews.

### Eligibility for each section of fever

If any member of household had fever within past one month, he or she was eligible for recent fever section. If a forest goer had fever, he or she was eligible for both recent and past fever sections. Therefore, more than one forest goer completed the past fever section if they were eligible. Past fever episode was any fever episode in the past occurred in residential village and working in forest. If there was any RDT testing experience during the past, it was explored for residential village and working in forest for forest goers.

### Data collection

Data were collected by PSI/Myanmar research team in October and November, 2019 with instruments developed by GEMS Program Team and PSI/Myanmar Research Department. For health service provider mapping, mapping tools were used to collect data from all the fixed (venue or facility based) and mobile health services within the village. For the household survey, a structured household survey questionnaire was used to collect demographic information of members, forest goer information and their knowledge of malaria transmission and prevention. Household socio-economic status information such as household assets, housing materials, drinking water source etc. was collected through a short version of equity tool [24]. For exploring health-seeking behaviors of any household members, experience of having a febrile illness during past one month and how a care was sought was asked in the survey. To provide an insight for differences in decision making based on fever locations, forest goers in every household were asked about how they received care during past fever episodes as well as RDT testing providers if there was any. All data collection tools were developed in English and translated into Myanmar. Face-to-face interviews were conducted in Myanmar language. Data was collected by tablets using C. S Pro version 7.1. Field data were uploaded to the server daily and data quality checks were done for completeness and consistency of the quantitative data as stated in the study protocol.

### Data preparation and variable selection

To find the association between choice of providers and potential predictors based on fever episodes and RDT testing, analyses were done for 5 scenarios; recent fever episode for any member of household, past fever episode in residence, that in forest for forest goers as well as RDT testing in residence and that in forest for forest goers. Dependent variable was a polychotomous variable reflecting the four provider alternatives: i. Private, semi-private and informal providers, ii. Public facility and health staff and iii. Community health worker or volunteer and iv. Outside providers-all type of health care providers located outside of the village boundary. The provider categorization was based on the common types of providers that patients seek care for malaria febrile illnesses in Myanmar. The outcome variable for fever had four categories (Public, Semi-private, CHV/CHW and Outside providers) and the outcome variable for RDT testing had three categories (Non-CHV/CHW, CHV/CHW and utside providers) depending on the choices that the respondents made for each occasion. The questionnaire and codebook were reviewed in order to identify questions and variables that would be useful in identifying choice of health care provider as well as potential predictors of their health-seeking practice.

Independent variables included in the analyses were characteristics such as age (continuous variable), gender (male and female), relationship with household head (household head himself or family member) and having correct knowledge of malaria transmission and prevention (correct and incorrect knowledge). In addition, access to health care providers, which was reflective of respondent’s accessibility to health care providers in their residential village, was also included (access to 1 to 3 providers and access to 4 and above providers). The minimum number of providers that respondents accessed to was 1 and the average number of providers in the villages was 3 in the study. Household socio-economic status (5 quintiles from poorest to richest) and education level of main income earner (illiterate,primary and secondary and above) were also treated as independent variables. Household socio-economic status was derived using a set of household assets questions [24]. Tool measures relative wealth of a household, in which household assets were transformed into a composite score and a cut-off used to derive five wealth quintiles [25]. The cut-offs applied were those from national quintiles, therefore, the wealth of studied households represented their relative wealth with reference to national wealth quintiles [26].

### Data analysis

Data cleaning, management and analyses were performed using STATA version 14 [27]. Percentages and 95% Confidence Intervals (CI) were computed for all categorical variables of interest. Means and standard deviations were calculated for all continuous variables. Bivariate regressions were done to investigate the bivariate relationship between each potential predictor variable and the outcome of interest which was the provider choice variable. Statistical significance was set as p = 0.05. To control the effect of confounding variables, multinomial logistic regressions were done. P value and 95% CI were used to interpret the findings.

### Multinomial logistic model

The multinomial logistic model used the following equation. J + 1 is the number of distinct categories in the dependent variable and assume that the category 0 is selected as the base category. Then the probabilities given by the multinomial logistic function are:

$${\text{p (Y = j) = }}\frac{{{\text{Exp (}}\beta^{\prime}_{j} {\text{x}}_{{\text{i}}} )}}{{1 + \sum\limits_{{{\text{k}} = 1}}^{{\text{J}}} {{\text{Exp (}}\beta^{\prime}_{k} {\text{x}}_{{\text{i}}} )} }}\quad {\text{for j = 1,}} \ldots {\text{, J and}}$$

$${\text{P}}\,(Y = 0) = \frac{1}{{1 + \sum\limits_{{{\text{k}} = 1}}^{{\text{J}}} {{\text{Exp}}\,{(}\beta \prime_{k} {\text{x}}_{{\text{i}}} )} }}\quad {\text{for}}\,{\text{the}}\,{\text{base}}\,{\text{category}}.$$

where $\beta^{\prime}_{j}$ is the vector of estimated coefficients for the j^th^ category and ${\text{x}}_{{\text{i}}}$ is the i^th^ case (row) of the data matrix.

The relative risk ratio for case i relative to the base category is:

$$\frac{{{\text{p}}_{{{\text{ij}}}} }}{{{\text{P}}_{{{\text{i}}0}} }} = {\text{Exp}}\,{(}\beta^{\prime}_{j} {\text{x}}_{{\text{i}}} )\quad for\,j = 1, \ldots ,J\,and\,i = 1, \ldots ,n$$

The first step in building the multivariable multinomial logistic model involved conducting simple multinomial model between each of the potential predictors, and the polychotomous choice of health service provider variable. Age and gender were added in the model with a priori belief that they might have influence on provider choice as well as they found to be associated with malaria treatment-seeking behaviour [16, 33, 51]. For other variables, those significantly associated with the outcome (p < 0.05) in bivariate analyses were considered for inclusion in the multivariate model. However, to avoid highly correlated predictor variables, two-way correlations between the predictor variables were assessed using Pearson’s correlation coefficient. Relative risk ratios (RRR) and their 95% confidence intervals (CI) were then computed for all variables in the final model. Model goodness-of-fit was assessed using the Stata command mlogitgof and post-test diagnostics were done using mlogtest [28, 29].

### Ethical statement

Ethical approval was obtained from Population Services International Research Ethics Board with the approval number 40.2019, and Institutional Review Board-1 of Ministry of Health and Sports Myanmar with the approval number IRB 1/2019–2.

## Results

Table 1 showed the characteristics of household members who had a recent fever episode and that of forest goers who reported that they normally sought care from a provider during their previous fever episodes. There was a total of 307 individuals (which comprised 66.1% non-forest goers and 33.9% forest goers) who reported that they had fever within the past month. Among different age groups, “5 − 18 years” was the largest group with 31.6%. For previous fever episodes, there were 509 forest goers who reported about their previous fever episodes, of which majority (44%) were middle-aged individuals of 25–40 years. Overall, the following characteristics were similar for both recent and previous fever episodes. About 50% were in the poorest two quintiles. Majority were male and they were accessible to 3 to 4 providers in their village, having high level of correct malaria transmission knowledge but not knowing much about correct malaria prevention. The education level of main income earner in their households (above 63%) were mainly illiterate or had primary education.

**Table 1:** Characteristics of patients seeking a treatment for recent fever and characteristics of forest goers who reported previous fever episodes

Characteristic of patients seeking a treatment for recent fever (N = 307)	Categories	n	Percentage (%)
Age	< 5 yrs	50	16.3
	5–18 yrs	97	31.6
	19–24 yrs	18	5.9
	25–40 yrs	73	23.8
	41–64 yrs	62	20.2
	65 yrs and above	7	2.3
Sex	Male	157	51.1
	Female	150	48.9
National quintiles	Poorest quintile	74	24.1
	2nd quintile	71	23.13
	Middle quintile	94	30.62
	4th quintile	58	18.89
	Best-off quintile	10	3.26
Access	1 to 2 providers	61	19.87
	3 to 4 providers	213	69.38
	5 to 6 providers	33	10.75
Education level of main income earner	Illiterate and primary education	196	63.84
	Secondary and high school education	106	34.53
	Above high school education	5	1.63
Relationship with HH head	Self	56	18.24
	Family member	251	81.76
Malaria transmission knowledge level	Correct	214	80.15
	Incorrect	53	19.85
Malaria prevention knowledge level	Correct	90	33.71
	Incorrect	177	66.29
Forest goers	Yes	104	33.88
	No	203	66.12

*Characteristics of forest goers who reported previous fever episodes (N* = *509)*			
Characteristic	Categories	n	Percentage (%)
Age	< 5 yrs	2	0.4
	5–18 yrs	11	2.2
	19–24 yrs	55	10.8
	25–40 yrs	224	44
	41–64 yrs	203	39.9
	65 yrs and above	14	2.8
Sex	Male	419	82.3
	Female	90	17.7
National quintiles	Poorest quintile	137	26.92
	2nd quintile	112	22
	Middle quintile	142	27.9
	4th quintile	94	18.47
	Best-off quintile	24	4.72
Access	1 to 2 providers	163	32.02
	3 to 4 providers	279	54.81
	5 to 6 providers	67	13.16
Education level of main income earner	Illiterate and primary education	323	63.46
	Secondary and high school education	176	34.58
	Above high school education	10	1.96
Relationship with HH head	Self	297	58.35
	Family member	212	41.65
Malaria transmission knowledge level	Correct	381	74.85
	Incorrect	128	25.15
Malaria prevention knowledge level	Correct	162	31.83
	Incorrect	347	68.17

Table 2 described the types of providers that the household members consulted during their recent febrile illness and forest goers did so during their previous febrile illness. During the previous illness, forest goers were asked where they usually sought care for fever and RDT testing while they were back in residing villages and while working in forest. These providers were further stratified as “within their village” and “outside their village”. The providers were considered “within their village” if they were located within village geographic boundary and mapped during the survey. Those located outside of village geographic boundary were considered as outside providers.

**Table 2:** Choice of providers for recent fever, previous fever and previous RDT testing

	Recent fever (N = 222)		Previous fever							
			Fever in village (N = 319)		Fever in forest (N = 316)		RDT in village (N = 286)		RDT in forest (N = 280)	
Provider Types	n	%	n	%	n	%	n	%	n	%
*Within village*	**139**	**62.6**	**242**	**75.9**	**237**	**75.0**	**233**	**81.5**	**232**	**82.9**
Public facility	13	5.9	8	2.5	8	2.5	10	3.5	10	3.6
Public basic health staff	21	9.5	32	10.0	31	9.8	29	10.1	28	10.0
Private facility	0	0.0	0	0.0	0	0.0	0	0.0	0	0.0
Semi-private providers	19	8.6	22	6.9	27	8.5	3	1.0	2	0.7
Pharmacy	5	2.3	11	3.4	4	1.3	4	1.4	2	0.7
Community health volunteer or worker	81	36.5	169	53.0	167	52.8	187	65.4	190	67.9
*Outside Village*	**83**	**37.4**	**77**	**24.1**	**79**	**25.0**	**53**	**18.5**	**48**	**17.1**
Public facility	16	7.2	1	0.3	1	0.3	5	1.7	3	1.1
Public basic health staff	15	6.8	26	8.2	23	7.3	14	4.9	13	4.6
Private facility	26	11.7	30	9.4	34	10.8	22	7.7	19	6.8
Semi-private providers	2	0.9	6	1.9	7	2.2	6	2.1	7	2.5
Pharmacy	11	5.0	1	0.3	1	0.3	0	0.0	0	0.0
Community health volunteer or worker	13	5.9	13	4.1	13	4.1	6	2.1	6	2.1

The bold values are the number and % providers that are located inside the village and those outside the village

For both recent and previous fever illnesses, the respondents mostly relied on health providers located within their villages and the range being approximately between 62 and 82%. And their most-cited providers were community health workers or community volunteers. The range was between 36.5–67.9%. The results were more prominent in cases of RDT testing during previous febrile illness with more than 60%. In contrast, fewer respondents went to outside providers in both recent and previous fever illness with a range of 17–37%.

Table 3 shows the results of simple regression between individual characteristics of household members or forest goers (predictors) and different provider choices for treatment during recent and previous febrile illnesses.

**Table 3:** Predictors and their bivariate relationship with the different choice of providers among patients in recent febrile illness

Characteristic	Provider choice for recent fever					
	Public sector		Semi-private		Outside	
	RRR	(95% CI)	RRR	(95% CI)	RRR	(95% CI)
Age^a^	0.98^b^	0.95–1.00	0.99	0.97–1.01	0.98^b^	0.97–1.00
Non-forest goer	As reference					
Forest goer	0.81	0.33–1.98	0.92	0.34–2.5	0.94	0.49–1.79
Gender						
Female	As reference					
Male	1.43	0.64–3.21	0.57	0.22–1.47	1.31	0.72–2.38
Relationship with household head						
Self	As reference					
Family member	2.35	0.63–8.71	1.14	0.34–3.81	1.31	0.59–2.92
Socio-economic quintile						
Poorest quintile	As reference					
2nd poorest quintile	1.5	0.35–6.35	0.72	0.15–3.39	2.12	0.86–5.24
Middle quintile	3.33	0.97–11.43	0.89	0.23–3.45	2.12	0.91–4.96
2nd richest quintile	6^b^	1.42–25.39	5.4^b^	1.39–20.93	5.77^c^	2.03–16.38
Richest quintile	6	0.65–55.66	4.8	0.54–42.63	2.77	0.41–18.74
Education of main income earner						
Illiterate or primary education	As reference					
Secondary and above education	3.05^b^	1.32–7.07	2.18	0.84–5.67	2.52^b^	1.32–4.82
Malaria transmission						
Having incorrect knowledge	As reference					
Having correct knowledge	0.96	0.33–2.77	0.542	0.18–1.66	1.50	0.64–3.52
Malaria prevention						
Having incorrect knowledge	As reference					
Having correct knowledge	0.64	0.25–1.64	0.45	0.14–1.48	0.81	0.41–1.57
Access to no. of providers in the village						
Access to 1–3 providers	As reference					
Access to ≥ 4 providers	28.69^c^	6.41–128.45	4.35^c^	1.62–11.73	0.57	0.30–1.10

*Predictors and their bivariate relationship with the different choices of providers among forest goers in previous febrile illness that occured in residential village and forest*												
Characteristic	Provider choice for fever in village						Provider choice for fever in forest					
	Public sector		Semi-private		Outside		Public secto		Semi-private		Outside	
	RRR	(95% CI)	RRR	(95% CI)	RRR	(95% CI)	RRR	(95% CI)	RRR	(95% CI)	RRR	(95% CI)
Age ^a^	1	0.97–1.03	1.02	0.99–1.05	1.01	0.99–1.03	1	0.98–1.03	1.01	0.99–1.04	1.01	0.99–1.03
Gender												
Female	As reference											
Male	1.27	0.46–3.55	2.82	0.64–12.5	0.6	0.30–1.17	1.31	0.47–3.66	5.79	0.76–44.25	0.76	0.38–1.51
Relationship with household head												
Self	As reference											
Family member	1.09	0.54–2.21	0.62	0.27–1.41	0.94	0.54–1.64	1.01	0.49–2.06	0.39	0.15–0.99	0.99	0.57–1.71
Socio-economic quintile												
Poorest quintile	As reference											
2nd poorest quintile	3.18	1.02–9.91	2.17	0.57–8.22	1.96	0.88–4.39	5.55^b^	1.45–21.25	2.12	0.63–7.18	2.05	0.92–4.60
Middle quintile	1.2	0.35–4.16	2.5	0.74–8.45	1.57	0.73–3.38	2.42	0.59–9.84	1.87	0.59–5.93	1.63	0.76–3.51
2nd richest quintile	5.79^c^	1.76–19.1	7.96^c^	2.26–28	4.34^c^	1.85–10.2	10.37^c^	2.57–41.85	5.6^b^	1.66–18.88	5.11^c^	2.18–11.96
Richest quintile	44^c^	7.28–266.11	13.75^b^	1.51–124.99	1.96	0.17–23.25	74.67^c^	10.77–517.77	5.6	0.43–73.09	2	0.17–23.67
Education of main income earner												
Illiterate or primary education	As reference											
Secondary and above education	3.11^c^	1.52–6.35	1.75	0.83–3.72	1.47	0.85–2.55	2.05	0.92–4.6	1.76	0.81–3.82	1.49	0.87–2.58
Malaria transmission												
Having incorrect knowledge	As reference											
Having incorrect knowledge	1.93	0.80–4.64	0.71	0.33–1.56	0.85	0.47–1.52	1.61	0.69–3.75	0.66	0.30–1.46	1.01	0.56–1.82
Malaria prevention												
Having incorrect knowledge	As reference											
Having correct knowledge	1.24	0.61–2.52	0.50	0.21–1.23	0.75	0.41–1.34	1.27	0.62–2.60	0.53	0.22–1.31	0.71	0.39–1.27
Access to no. of providers in the village												
Access to 1–3 providers	As reference											
Access to ≥ 4 providers	6.38^c^	3.04–13.38	5.2^c^	2.37–11.38	0.21^c^	0.07–0.61	6.99^c^	3.28–14.86	3.22^c^	1.45–7.18	0.44	0.19–1.00

*Predictors and their bivariate relationship with the different choices of providers among forest goers for RDT testing in previous febrile illness that occured in residential village and forest*								
Characteristic	Provider choice for m-RDT testing in village				Provider choice for m-RDT testing in forest			
	Non-CHW/CHV (Public sector & Semi-private)		Outside		Non-CHW/CHV(Public sector & Semi-private)		Outside	
	RRR	(95% CI)	RRR	(95% CI)	RRR	(95% CI)	RRR	(95% CI)
Age ^c^	1	0.98–1.03	1.01	0.98–1.03	0.99	0.96–1.02	1.01	0.98–1.03
Gender								
Female	as reference							
Male	1.89	0.7–5.12	1.58	0.66–3.78	2.07	0.69–6.19	1.67	0.66–4.23
Relationship with household head								
Self	as reference							
Family member	0.85	0.44–1.62	0.68	0.37–1.26	0.79	0.40–1.55	0.79	0.42–1.46
Socio-economic quintile								
Poorest quintile	as reference							
2nd poorest quintile	1.78	0.61–5.15	0.73	0.3–1.8	1.53	0.52–4.54	0.8	0.32–2.01
Middle quintile	1.4	0.5–3.94	0.52	0.21–1.26	1.12	0.38–3.30	0.52	0.21–1.33
2nd richest quintile	4.64^c^	1.67–12.88	2.32^b^	1.02–5.27	4.57^c^	1.62–12.84	2.79^b^	1.2–6.46
Richest quintile	16.57^c^	3.37–81.46	3.41	0.63–18.47	16.86^c^	3.43–82.83	3.93	0.72–21.48
Education of main income earner								
Illiterate or primary education	as reference							
Secondary and above education	2.03	1.05–3.91	1.03	0.56–1.89	2.11	1.07–4.17	1.14	0.61–2.11
Malaria transmission								
Having incorrect knowledge	as reference							
Having correct knowledge	0.89	0.40–1.95	0.99	0.46–2.09	1.03	0.44–2.40	0.9	0.42–1.92
Malaria prevention								
Having incorrect knowledge	as reference							
Having correct knowledge	0.46^b^	0.22–0.96	0.6	0.31–1.15	0.47	0.22–1.01	0.73	0.38–1.39
Access to no. of providers in the village								
Access to 1–3 providers	as reference							
Access to ≥ 4 providers	5.08^c^	2.56–10.07	1.33	0.69–2.58	4.94^c^	2.44–10.00	1.48	0.76–2.88

CHW/CHV as base outcome

^a^continuous variable

^b^p < 0.05

^c^p < 0.01

With CHW and CHW as base outcome, age of household member had a significant negative association (p = 0.04) with the choice of public providers and those located outside of the village in recent febrile illness. Those in the second richest quintile had significant positive associations with choice of all different types of providers such as public providers (p = 0.02), semi and private providers (p = 0.02) and outside providers (p < 0.001) but the association between other wealth quintiles and provider choice were not statistically significant. Having an access to more than 4 providers in their village also had significant positive association with the choice of public providers (p < 0.001) and semi-private and private providers (p < 0.001), and main income earner’s education; those with secondary and above also had significant positive association with the choice of public providers and providers located outside with p = 0.01.

The results of bivariate regression analysis between forest goers’ characteristics and different provider choices for treatment when they got fever in the village and in the forest were presented in Table 3. In both situations, access to 4 or more providers inside the village had significant positive relationships with the choice of providers from public sector and that from private and semi-private sector while forest goers who had access to more than four providers in their village were less likely to receive treatment from the outside providers. The second richest quintile had a significant positive association with the choice of public providers, private and semi-private providers and those who resided outside the village. The richest quintile had significant positive relationship with choice of public providers and semi-private providers in fever that occurred in village and similar pattern was seen with choice of public providers for fever that occurred in forest. Most of the positive association between wealth quintiles and choice of providers were not statistically significant. The secondary and above education level was found to be associated with the use of providers from public sector with p value < 0.001.

The results of bivariate regression between characteristics of forest goers and their provider choice for malaria RDT testing during previous febrile illness while they were at the village and forest were presented in Table 3. Community health workers and volunteers were considered as base outcome in these models. For both situations, the richest, second richest quintile and access to more than four providers had significant positive association (p value < 0.001) with the choice of providers from non-CHW/CHW for RDT testing while the respondent with correct malaria prevention knowledge were likely (p value = 0.04) to choose providers from non-CHW/CHW for RDT testing in village. The second richest quintile had a significant positive association (p value < 0.05) with the choice of providers resided outside the study area while they tested for RDT in the village and forest. There were positive associations between 2nd poorest and middle wealth quintiles and choice of from non-CHW/CHW and negative associations between these two quintiles and choice of providers outside the village. But the results were not statistically significant.

Table 4 presents the results of the multinomial logistic regression analysis on predictors of different choice of providers among household members in recent febrile illness and forest goers in previous illness. With CHW/ CHV providers as the base outcome, it was seen that a unit increase in household member’s age would be less likely to use public health provider (RRR = 0.97; p value 0.03) for recent febrile illness. No significant difference was observed between the age and choice of semi-private providers and outside providers. Compared with those in the poorest wealth quintile, those from higher socioeconomic status households were more likely to use public providers, semi-private health provider and outside providers for recent febrile illness. Specifically, those from second richest quintiles showed strong significant association with choice of all types of providers compared to those in the poorest quintiles. Individuals who had access to more than 4 health providers in their village were more likely to use public health provider (RRR = 30.27; p value < 0.001), semi-private providers (RRR = 11.54; p value < 0.001) for recent febrile illness compared to those who were accessible to 1–3 providers. However, they were less likely to go to outside provider (RRR = 0.48; p value 0.07) compared to those who had access to 1–3 providers inside their village.

**Table 4:** Multivariable Multinomial Logistic Regression modeliInvestigating predictors of different choices of providers among patients in recent febrile illness

Characteristic	Provider choice for recent fever					
	Public sector		Semi-private		Outside	
	RRR	(95% CI)	RRR	(95% CI)	RRR	(95% CI)
Age ^a^	0.97^b^	(0.94–1.00)	0.98	(0.95–1.01)	0.98	(0.96–1.00)
Gender						
Female	As reference					
Male	1.16	(0.42–3.22)	0.3	(0.09–1.03)	1.15	(0.58–2.29)
Socio-economic quintile						
Poorest quintile	As reference					
2nd poorest quintile	1.17	(0.21–6.70)	0.31	(0.03–3.63)	2.17	(0.76–6.15)
Middle quintile	4.77	(0.91–25.16)	1.85	(0.33–10.34)	1.96	(0.77–5.01)
2nd richest quintile	12.85^c^	(1.93–85.41)	17.93^c^	(2.77–116.05)	8.39^c^	(2.54–27.76)
Richest quintile	3.24	(0.28–38.11)	3.94	(0.34–46.12)	4.81	(0.62–37.31)
Access to no. of providers in the village						
Access to 1–3 providers	As reference					
Access to ≥ 4 providers	30.27^c^	(6.13–149.41)	1.54^c^	(2.78–47.88)	0.48	(0.22–1.07)
Malaria transmission						
Having incorrect knowledge	As reference					
Having correct knowledge	0.36	(0.09–1.40)	0.17^b^	(0.04–0.70)	1.35	(0.54–3.36)
Constant	0.06^b^	(0.01–0.51)	0.27		0.66	(0.21–2.05)

*Multivariable Multinomial Logistic Regression model investigating predictors of different choice of providers among forest goers in past febrile illness that occur in the village and forest*												
	Provider choice for fever in village						Provider choice for fever in forest					
	Public		Semi-private		Outside		Public		Semi-private		Outside	
	RRR	95%CI	RRR	95%CI	RRR	95%CI	RRR	95%CI	RRR	95%CI	RRR	95%CI
Age ^a^	1.02	(0.99–1.05)	1.04^b^	(1.01–1.07)	1.01	(0.98–1.03)	1.03	(0.99–1.06)	1.03^b^	(1.00–1.07)	1.01	(0.99–1.04)
Gender												
Female	As reference											
Male	1.24	(0.38–4.06)	3.92	(0.80–19.17)	0.55	(0.27–1.12)	1.03	(0.32–3.30)	7.06	(0.88–56.51)	0.76	(0.37–1.57)
Socio-economic quintile												
Poorest quintile	As reference											
2nd poorest quintile	2.84	(0.86–9.36)	2.03	(0.51–8.00)	1.99	(0.88–4.52)	5.19^b^	(1.28–21.04)	2.06	(0.59–7.18)	2.06	(0.91–4.65)
Middle quintile	0.94	(0.26–3.46)	2.2	(0.62–7.82)	1.64	(0.74–3.61)	1.89	(0.44–8.14)	1.79	(0.55–5.91)	1.71	(0.78–3.75)
2nd richest quintile	4.21^b^	(1.20–14.82)	6.35^c^	(1.71–23.56)	4.71^c^	(1.95–11.37)	7.57^c^	(1.76–32.60)	5.00^b^	(1.43–17.52)	5.36^c^	(2.25–12.76)
Richest quintile	20.21^c^	(3.05–133.88)	6.66	(0.69–64.54)	2.77	(0.22–34.70)	37.18^c^	(4.83–286.02)	2.99	(0.22–40.64)	2.27	(0.19–27.83)
Access to no. of providers in the village												
Access to 1–3 providers	As reference											
Access to ≥ 4 providers	5.43^c^	(2.36–12.51)	5.45^c^	(2.31–12.87)	0.18^c^	(0.06–0.54)	6.00^c^	(2.56–14.09)	3.45^c^	(1.45–8.18)	0.41^b^	(0.17–0.95)
Education of main income earner												
Illiterate or primary education	As reference											
Secondary and above education	2.89^b^	(1.27–6.57)	1.77	(0.76–4.12)	1.56	(0.86–2.84)	3.39^c^	(1.44–7.96)	1.78	(0.77–4.12)	1.61	(0.89–2.91)
Constant	0.00^c^	(0.00–0.06)	0.00^c^	(0.00–0.02)	0.21	(0.04–1.09)	0.00^c^	(0.00–0.03)	0.00^c^	(0.00–0.03)	0.11^c^	(0.02–0.57)

*Multivariable Multinomial Logistic Regression model investigating predictors of different choice of providers among forest goers for RDT testing in past febrile illness that occured in the village and forest*								
	Provider choice for m-RDT testing in village				Provider choice for m-RDT testing in forest			
	Non-CHW/CHV		Outside		Non-CHW/CHV		Outside	
	RRR	95%CI	RRR	95%CI	RRR	95%CI	RRR	95%CI
Age ^a^	1.02	(0.99–1.05)	1.01	(0.99–1.04)	1.01	(0.98–1.04)	1.01	(0.98–1.04)
Gender								
Female	As reference							
Male	2.05	(0.68–6.18)	1.52	(0.61–3.76)	1.91	(0.58–6.29)	1.62	(0.61–4.25)
Socio-economic quintile								
Poorest quintile	As reference							
2nd poorest quintile	1.99	(0.65–6.06)	0.74	(0.30–1.84)	1.65	(0.53–5.08)	0.8	(0.32–2.01)
Middle quintile	1.51	(0.51–4.45)	0.54	(0.22–1.32)	1.15	(0.38–3.49)	0.54	(0.21–1.37)
2nd richest quintile	4.55^c^	(1.55–13.37)	2.46^b^	(1.07–5.67)	4.28^b^	(1.44–12.66)	2.85^b^	(1.22–6.69)
Richest quintile	9.92^c^	(1.81–54.51)	3.51	(0.61–20.11)	9.81^b^	(1.79–53.70)	3.64	(0.63–20.92)
Access to no. of providers in the village								
Access to 1–3 providers	As reference							
Access to ≥ 4 providers	4.68^c^	(2.22–9.89)	1.23	(0.61–2.51)	4.19^c^	(1.94–9.06)	1.36	(0.66–2.79)
Malaria prevention								
Having incorrect Knowledge	As reference							
Having correct Knowledge	0.39^b^	(0.18–0.87)	0.56	(0.28–1.10)	0.41^b^	(0.18–0.94)	0.68	(0.35–1.34)
Constant	0.02^c^	(0.00–0.16)	0.16^b^	(0.03–0.70)	0.04	(0.01–0.29)	0.12	(0.03–0.59)

CHW/CHV as base outcome

^a^continuous variable

^b^p < 0.05

^c^p < 0.01

## Discussion

Forest goers’ choice of provider for health-seeking for fever was found to be associated with their access to number of providers in their residential area as well as their socio-economic status. The age and knowledge of the forest goers’ were negatively associated with the decision to choose different types of providers.

This study found that forest goers’ access to higher number of providers could have motivated them to seek care from such nearby providers because they mostly resided in rural villages and could not travel far when they fell sick. In addition, their work nature did not generally allow them to stay away from work since they were paid daily. In addition, their socio-economic status seemed to influence their health-seeking behaviour from providers. Self-medication and home treatment were found to be common in these populations [30–32]. Age was found to be a predictor for providers’ choice and the association was mixed for different occasions. However, the effect was not very strong as in other predictors because the reason for such finding could be that the study respondents were actively involved in forest related jobs and they were working age group individuals [33]. There was a negative association of correct knowledge on malaria transmission and prevention with choice of providers was found in our study. It indicated that the knowledge did not seem to translate into practice in this particular population because malaria has been popular among them and they already had knowledge related to transmission [32].

Studies from Laos [34], Cambodia [35–37] and Bangladesh [38] show that economic constraints influenced health-seeking behaviour for malaria significantly, causing health service underutilization and delay in malaria treatment seeking in people with lower income. In a study in Myanmar [16], family income was associated with malaria treatment-seeking behaviour. Financial incapability was a major barrier to get malaria treatment [39, 40]. Similarly, a study done in Ghana also found a negative association between perceived relative economic status (a proxy measure for economic status) and the use of herbal/traditional treatment as the first response to malaria, meaning that the lower an individual’s economic status, the higher the chance of using alternative treatment for malaria and vice versa [41]. The findings in the study were similar to the above findings that those in richer wealth quintiles were more likely to seek care from a provider either inside the village or outside the village compared to those in poorer quintiles.

Regarding proximity, the proximity of the household to the health center served as an important factor in the decision making of malaria patients to utilize health service of a health facility [16, 34, 42]. A study in Ethiopia showed that the decision of malaria patients to seek treatment at a health facility was dependent on the distance from home [42] and similar findings were found in Cambodia [36, 37]. Similarly, in Myanmar, the long distances between the malaria patients’ home and local health facilities made them reluctant to seek treatment there [16, 33, 43]. This was in contrast to studies carried out in Thailand [44] and Sri Lanka [45]. A possible explanation was that in Thailand and Sri Lanka, patients had long distances but easier access to convenient forms of travel than was the case in Ethiopia [42, 46] and Myanmar [16]. The findings from this study were similar to Ethiopian, Cambodian and previous Myanmar studies that distance seemed to matter in seeking care from a provider and being proximal to 4 and more number of providers in the village made patients seek care from those providers.

With regards to treatment for fever relief, in Asian countries like Thailand [31], Sri Lanka [45] and India [47], malaria patients treats on their own with the use of left-over drugs at home or drugs from a convenience shop or drug shop as a primary treatment. A study in Myanmar showed that nearly half of respondents did not seek proper treatment and utilized untrained informal health care providers such as quacks, traditional healers, seeking assistance from drug vendor/shops and self-medication after the onset of malaria symptoms [48]. Research studies indicated that patients did not seek treatment within 24 hours because of the high cost of diagnosis and treatment, and accessibility to health care facilities [16, 31, 42, 49, 50]. In these studies, the delay in seeking treatment was associated with affordability, distance from health facilities including availability of the health care service. Similar findings were found in this study that forest goers and their family members’ choice of providers were determined by socio-economic status and having more providers in their village [15, 16, 30, 31, 41, 42, 48–50]. In cases of knowledge of malaria prevention and transmission, almost all of the forest goers in a Thailand study were aware that malaria was transmitted by mosquitoes and bed nets were used to prevent malaria [32]. Similarly, it was found that forest-goers in GMS countries often had knowledge of malaria transmission [20]. Other studies in Vietnam and Myanmar found that the forest goers and individuals at risk of malaria understood that malaria was transmitted by mosquitoes [30, 51] and prevented by mosquito nets [51]. These findings were consistent with the findings from the current findings that 80.2% of forest goers had correct malaria transmission knowledge and 33.9% had malaria prevention knowledge. However, having correct knowledge seemed not to translate into seeking proper malaria febrile illness care. Low level of correct knowledge of malaria transmission and prevention in the community was reported in a previous study done in Myanmar [10]. In another study in Myanmar, although the majority of the respondents reported that malaria could be transmitted from person-to-person through mosquito bites and could be prevented by the use of mosquito net, delayed diagnosis and treatment of febrile illness were demonstrated [52].The studies done in Ethiopia [42] and southern Ghana [53] found that knowledge of respondents had no association with malaria treatment-seeking. Nevertheless, the study done in Myanmar has shown that elderly age [51] and poor knowledge about malaria were associated with poor treatment-seeking behaviour for febrile illness [43, 51]. Several previous studies had also found that those who had greater malaria knowledge score were over two times more likely to seek care from trained providers than those without sufficient knowledge [54–56]. Such findings were in contradiction with the findings from this study that the knowledge was not positively associated with practice in heath-seeking.

In terms of policy and practice implications, it was important to ensure that forest goers and their household members received a RDT testing and proper treatment within 24 hours of symptoms onset. Therefore, there was a need to educate the significance of testing and obtaining treatment within 24hours onset of fever as well as to encourage these forest goer households to adopt proper health-seeking from trained and qualified providers in their geographic areas. Creating enabling environment such as subsidized rates for care and providing regular training and equipment to those nearby providers should be prioritized by government and programme. Engagement with these providers at every level of health system was critical in order to focus the preference of target population in elimination efforts. Since the forest goers could not be reachable by conventional passive case detection and management activities, an active case detection and case management strategy that is tailored to the nature of forest goers might be a possible solution. A multiphase strategy aimed to do active case detection using mobile malaria workers worked well for such populations in Cambodia [57]. In Myanmar, an ongoing programme strategy that was particularly engaging private non-formal sector to provide high quality malaria care and data reporting was proved to be effective to provide quality testing and case management for high-risk populations. Therefore, such approaches could be continued until all high risk forest goer populations became no more high risk groups [58, 59] Future research could focus on quality assessment of the care obtained from these nearby providers as well as from the costing aspect. Operational research on evaluation of a variety of engagement strategies with forest goers could also be another possibility to inform the optimal design for programmes.

The study has both strengths and limitations. First, this is the first study in Myanmar with an only focus on forest goer population that are resided proximity to forests in malaria endemic regions across the countries. Second, unlike the past literature, the study could explore not only the health-seeking behaviors of recent febrile illness but also that of previous ones particularly while they were in their village and working in forest. On the other hand, the study collected self-reported illness and health-seeking behaviours which could have recalled bias but the training of enumerators and careful selection of variables for data analysis could have minimized the effect of bias. Another point to note is that the respondents were those physically present at their houses when the study was conducted and not those working in forest. However, it was believed that additional inclusion of those working in forest would not change the present results. The study findings may be generalizable to forest goers in Myanmar, but care should be taken when comparing this study results with those in other countries because of the use of own operational definition of forest goers.

## Conclusion

The study highlighted that in forest goer households, forest goers and their family members preferred nearby providers and the decision to choose providers seemed to be influenced by their access to number of nearby providers in their geographic areas and socio-economic status when they sought care from a provider regardless of fever occurrence location. It is important that the national programmes consider involving these nearby providers in elimination efforts in a consistent manner. This will ensure that the target population is provided the standardized care as to the National Guidelines from the providers that they favoured. Future research could focus on quality assessment of the care obtained from these nearby providers, costing aspect and evaluation of different engagement strategies with forest goers.

## Data Availability

The quantitative dataset will be available from the corresponding author on reasonable request.
